# Development and Validation of Environmental DNA (eDNA) Markers for Detection of Freshwater Turtles

**DOI:** 10.1371/journal.pone.0130965

**Published:** 2015-07-22

**Authors:** Christina M. Davy, Anne G. Kidd, Chris C. Wilson

**Affiliations:** 1 Wildlife Preservation Canada, Guelph, Ontario, Canada; 2 Trent University, Peterborough, Ontario, Canada; 3 Ontario Ministry of Natural Resources and Forestry, Peterborough, Ontario, Canada; Central Michigan University, UNITED STATES

## Abstract

Environmental DNA (eDNA) is a potentially powerful tool for detection and monitoring of rare species, including threatened native species and recently arrived invasive species. Here, we develop DNA primers for a suite of nine sympatric freshwater turtles, and use it to test whether turtle eDNA can be successfully detected in samples from aquaria and an outdoor pond. We also conduct a cost comparison between eDNA detection and detection through traditional survey methods, using data from field surveys at two sites in our target area. We find that eDNA from turtles can be detected using both conventional polymerase chain reaction (PCR) and quantitative PCR (qPCR), and that the cost of detection through traditional survey methods is 2–10X higher than eDNA detection for the species in our study range. We summarize necessary future steps for application of eDNA surveys to turtle monitoring and conservation and propose specific cases in which the application of eDNA could further the conservation of threatened turtle species.

## Introduction

The use of environmental DNA (eDNA) for species detection and monitoring is becoming more common, with an increasing number of studies dedicated to testing and applying these methods [1]. Because organisms shed small amounts of DNA into their habitat (for example, in feces, shed skin cells, saliva, or other secretions), sampling eDNA from the environment rather than directly from the donor organism provides an option for rapid detection of species without physical capture or visual confirmation [1, 2]. Recent work with fish, amphibians and squamates demonstrates that eDNA extracted from water samples can be a powerful detection tool for a variety of aquatic species [3, 4, 5].

Surveys based on eDNA sampling have potential for applied conservation through detection of rare or cryptic species that may be overlooked with traditional survey methods [1]. Species of conservation concern often fall into this category due to low population numbers, and eDNA surveys have facilitated detection of a range of threatened taxa ranging from eastern hellbenders (*Cryptobranchus alleganiensis*) to long-finned pilot whale (*Globicephala melas*) and European eel (*Anguilla anguilla)* [6, 7, 8]. Exotic and invasive species are also typically rare at their expanding range margins, and eDNA surveys can monitor the spread of invasive species. Current examples include surveillance for Asian Carp in the Laurentian Great Lakes [4] and detection of Burmese pythons in waterways in Florida [5].

Given the potential applications of eDNA for detection of rare or cryptic species, it is surprising that it has not yet been applied to turtles in aquatic systems. Turtles are the most threatened group of vertebrates, with approximately half the extant species considered of conservation concern [9]. Visual and/or physical detection rates for turtle species vary substantially among sites and species, as well as among survey methods [10, 11]. Rare species can be particularly difficult to detect with traditional methods, especially in turbid waterways [12, 13]. Detection of eDNA could provide a more rapid, cost-effective, and potentially more sensitive survey tool to identify areas where turtle species are present, which can then be prioritized for intensive trapping surveys.

Detection of eDNA may not be equally effective for all taxonomic groups. Some organisms may shed less tissue into their environment than others, and habitat preferences and behaviour may limit accumulation and detectability of eDNA. For example, recent studies have shown both successes and limitations to the detection of reptiles in water [5, 14]. No turtle eDNA was detected in a survey of aquatic species in a set of European ponds within the range of the European Pond Turtle (*Emys orbicularis;* [15]), although this study did not set out to specifically target turtles. Universal primers used in a metagenomic analysis failed to detect eDNA from a green sea turtle (*Chelonia mydas*) in a large mesocosm [14], but targeted PCR-based amplification of the same water samples with species-specific primers was successful, suggesting that a targeted primer panel might be able to detect turtles in natural aquatic systems.

Here, we test whether eDNA can be an effective tool for detection of freshwater turtles. We develop and test a species-specific primers to target the full complement of turtle species that occur in Canada. Our primers target species that are native and exotic to our study area, including several species of global conservation concern. We validate the use of these primers to detect turtle eDNA in controlled water samples. We also conduct a preliminary assessment of turtle eDNA detection in the field by testing eDNA detection of turtles in a sample from a small pond to determine the appropriateness of conducting rigorous field trials in the future. Finally, we provide a preliminary cost comparison of eDNA surveys and traditional survey methods for freshwater turtles, based on a mark-recapture data-set from our study area.

## Materials and Methods

### Target study area

We targeted the eight turtle species native to Canada and sympatric within their Canadian range, as well as one recently established exotic species (listed in Table 1). Samples of eDNA from native species were collected from captive individuals (see below), and we also tested a single field sample from a small, man-made outdoor pond at Scales Nature Park in Orillia, Ontario. The pond is located at N44.561973, W-79.440809, and is approximately 3.5 m in diameter and approximately 1 m deep. It contains 6 red-eared sliders (*Trachemys scripta)* which are prevented from dispersing by a fence placed approximately 2m from the edge of the pond. No permits were required to collect water from aquaria at the two animal care facilities from which we sampled.

**Table 1:** Identity and conservation status of the nine target species. Global conservation status is based on assessment by the International Union for the Conservation of Nature (IUCN, [37;41–48]); conservation status within Canada is based on assessment by the Canadian Committee on the Status of Endangered Wildlife in Canada (COSEWIC, [49–56]). EN = endangered; THR = threatened; LC = Least Concern; SC = Special Concern; NA = not assessed.

Species	Native to Canada?	IUCN status	COSEWIC status
Blanding’s Turtle *(Emydoidea blandingii)*	Native	EN	EN/ THR*
Spotted Turtle (*Clemmys guttata*)	Native	EN	EN
Wood Turtle *(Glyptemys insculpta)*	Native	EN	THR
Painted Turtle (*Chrysemys picta)*	Native	LC	EN/SC/LC**
Northern Map Turtle *(Graptemys geographica)*	Native	LC	SC
Eastern Musk Turtle (*Sternotherus odoratus*)	Native	LC	SC
Snapping Turtle (*Chelydra serpentina*)	Native	LC	SC
Eastern Spiny Softshell (*Apalone spinifera*)	Native	LC	THR
Red-eared Slider (*Trachemys scripta*)	Exotic	LC	NA

* assessed as two Designatable Units [48]

** assessed as three Designatable Units [49]

### DNA extraction for positive controls

We obtained blood samples for all nine target species from the Royal Ontario Museum, Toronto, Canada. Tissue (blood) samples used in this study were collected under permits from the Ontario Ministry of Natural Resources and Forestry authorizing field work under the Ontario Endangered Species Act and Fish and Wildlife Act. Work with wild animals was approved under Animal Use protocol 2010–14 from the Animal Care Committee of the Royal Ontario Museum.

DNA from two individuals of each species was extracted from FTA cards (Whatman Inc.) using standard methods for extraction of DNA from nucleated blood [16]. We used these samples to confirm primer specificity and to create standards of known DNA concentration for quantitative polymerase chain reactions (qPCR).

### eDNA sample collection

To test whether turtle eDNA could be successfully amplified from water samples, we obtained 1L water samples from aquaria at Scales Nature Park, Orillia, Ontario, and from the Kawartha Turtle Trauma Centre, Peterborough, Ontario. Because all samples were taken from captive turtles kept in authorized animal care facilities, no additional authorizations were required to collect these water samples. We used 1L glass jars or plastic Nalgene bottles to collect water samples from tanks which had each contained one of the nine target species for a minimum of 24 hours. The relative water content and turtle biomass in each tank is listed in Table 2. For *Chelydra serpentina*, we also obtained multiple 1L water samples from tanks with high turtle:water concentrations and a tank with a lower turtle:water concentration. We conducted a preliminary test of our ability to detect turtle eDNA in natural habitats by sampling water from an outdoor pond at Scales Nature Park that contained red-eared sliders (*Trachemys scripta*; described above). We sampled by standing on the shore and reaching out to fill a 1L sterile Nalgene container with surface water. We carried a field control (sterile bottle filled with ddH_2_O) which was also filtered and tested for turtle DNA, to control for potential contamination of samples during work at the field site and aquaria.

**Table 2:** Species-specific primers targeting the mitochondrial cytochrome oxidase subunit I (COI) gene in the nine target species of turtle, showing fragment length (in base pairs; bp); annealing temperature for PCR (T_A_) and the biomass of turtle relative to water in each tested sample, and whether eDNA was detected through PCR or qPCR (Y = yes for all replicates; N = no;— = not tested).

						eDNA detected?	
Species	Primer	Sequence (5’-3’)	bp	T_A_	Target biomass in sample (g/L)	PCR	qPCR
*Emydoidea blandingii*	*CO1-EBl-01-F*	ATCATAATCTTCTTCATAGTC	216	56	12.50	Y	—
	*CO1-EBl-01-R*	AGTTTCCAGCTAGTGGTGGA					
*Clemmys guttata*	*CO1-CGu-01-F*	GCCATTAATAATCGGGGCACCG	227	65	133.35	Y	Y
	*CO1-CGu-01-R*	GAATTGAAGATACACCAGCCAAA					
*Glyptemys insculpta*	*CO1-GIn-02-F*	GCCAGTCATAATCGGTGGA	155	62	1.88	Y	—
	*CO1-GIn-02-R*	CTGCTCCTGCTTCAACCCCT					
*Chrysemys picta*	*CO1-CPi-01-F*	GAAATTGACTCGTACCAATG	230	60	3.18	Y	—
	*CO1-CPi-01-R*	CACCCCTGCTAAGTGGAGAG					
*Graptemys geographica*	*CO1-GGe-01-F*	GTTATTATTGCTCTTAGCATC	202	56	375.00	Y	—
	*CO1-GGe-01-R*	GGCTGGAGATTTTATGTTAA					
*Sternotherus odoratus*	*CO1-SOd-01-F*	CGCCTGAGCAGGCATAATTG	299	65	0.65	Y	—
	*CO1-SOd-01-R*	CTGCACCTGCTTCAATTCCA					
*Chelydra serpentina*	*CO1-CSe-01-F*	TGTTATAATTGGGGGCTTTGGA	197	60	248.00	Y	Y
	*CO1-CSe-01-R*	GAGCTATGTTTCCAGATAGT			10.71	Y	N
*Apalone spinifera*	*CO1-ASp-02-F*	CATCTGGCCGGAGTATCGTC	231	65	150.00	Y	—
	*CO1-ASp-02-R*	GTCTCCACCTCCTGAGGGAT					
*Trachemys scripta*	*CO1-TSc-01-F*	GGGAACTGACTCGTGCCATTA	178	65	~ 3.75	Y	—
	*CO1-TSc-01-R*	GGGCTAAATTTCCGGCTAAT					

Water samples were kept at 4°C after collection and filtered within 24 hours. We disinfected all lab equipment prior to filtering with a 10% bleach solution, and rinsed with tap water. We filtered samples using 1.2μm Whatman TM 47mm GF/C glass microfiber filters (http://www.whatman.com), and filtered 1L of double-distilled water (ddH_2_O) before and after each sample as negative pre- and post-filter controls. Filters were stored for up to 2 weeks at -80°C before DNA extraction.

DNA was extracted from whole filters following published best practices [17], using the DNeasy Tissue and Blood Kit (Qiagen, Inc.). We added extra lysis buffer (100μL) as required, and conducted the final elution at 65°C, using a low-EDTA TE buffer (10 mM Tris-HCl, 0.1 mM EDTA, pH 8.0) for elution in place of regular TE buffer. The pre- and post-filter controls were included in each PCR reaction to control for potential lab contamination.

### eDNA amplification

We used both conventional polymerase chain reaction (PCR) and quantitative PCR (qPCR) to amplify turtle DNA from our tissue and environmental samples. All PCR and qPCR cocktail preparation was carried out in a Mystaire bench top fume hood and UV cabinet, using 30 min of UV sterilization of tools and reagents before setting up each reaction. Using our positive control DNA, we first tested three primer sets on genomic DNA from all 9 species to amplify segments of the mitochondrial cytochrome c oxidase subunit I (COI) gene: universal primers (LCO1490 and HCO2198; [18]), fish primers (FishF1 and FishR1; [19]) and reptile primers (AmphF2 and AmphR2; [20]), following previously published PCR conditions for each primer pair [18, 20] with 40 cycles per reaction. Both the Fish and Amph primer sets have been used in previous studies to amplify DNA from several turtle species [20]. We visualized the amplified DNA on a 1.5% agarose gel using SybrGreen, and sequenced the amplicons to confirm species identity. We quantified amplified DNA and diluted it in ddH_2_O to create quantitative PCR standards for each species, ranging from 10^6^ to 10^0^ DNA copies/μL.

We designed species-specific primers targeting the COI gene for each of the nine target species (Table 2). CO1 sequences for the target species were downloaded from the Barcode of Life (BOLD) database, aligned, and trimmed to isolate the target region. For each species we used Primer3 [21] to create sets of potential primer pairs. We then used BLAST searches to confirm primer specificity, discarding any primer pairs that returned non-target sequences of any other species. Finally, further manual adjustments were made to minimize similarity between primers and the binding region in non-target turtle species, based on the downloaded CO1 sequences. Where possible we ensured that each primer contained an 18–25 bp region with a minimum of 2–3 mismatches with all non-target Canadian species (S1 Table). CO1 shows low variation within species and mtDNA is not highly variable in many North American turtles (e.g. [22, 23]). These primers are therefore likely to work for individuals throughout each species’ range, although we were not able to test this explicitly with isolates representing geographic variation.

Each 15 μL reaction included 1 unit of Promega 5X PCR buffer, 2 μg of bovine serum albumin, 2mM MgCl2, 0.2mM dNTPs, 0.2 μM each of forward and reverse primer, 0.025U of Promega *Taq* and 12 ng of DNA. Cycling parameters included an initial denaturation step at 95°C for 3 min; 35 cycles of 94°C for 45 s, the primer-specific annealing temperature for 45 s (see Table 2), and 72°C for 1 min; and ending with a final extension step of 72°C for 10 min. We tested each primer pair with DNA extracted from each known species’ tissues to determine species specificity and ensure that the primers did not amplify co-occurring, non-target species. We also used these primers to amplify extracted eDNA from aquaria and field samples (6 replicates per species·sample), visualizing amplified products on 1.5% agarose gels. To confirm species identity of the amplified products, amplified product was purified using Exonuclease I and Antarctic phosphatase (New England BioLabs), then sequenced in both directions using BigDye Terminator v3.1 chemistry (Applied Biosystems) on an ABI 3730 sequencer. Sequences were proofread using Sequencher v. 5.1 (Gene Codes Inc.), and compared with reference taxon sequences through a BLAST search.

These novel species-specific primers were also used for qPCR amplification of turtle eDNA from two species (*Ch*. *serpentina* and *Clemmys guttata*) for whom we had eDNA samples from tanks. These eDNA samples were run at dilutions of 1:10, 1:20 and 1:30 to test for inhibition. Each qPCR reaction included 10μL (1X) Power SYBR Green Master Mix (Life Technologies), 0.2 μM of forward and reverse primers and 5 μL of template eDNA, with ddH_2_0 added to reach total volume of 20μL. Reactions were run on a StepOnePlus Real-Time PCR System (Applied Biosystems). Cycling conditions included denaturation at 95°C for 10 min, following by 40 cycles of denaturation at 95°C for 15 s and copy replication at 60°C for 1 min. Species identity of qPCR products was assessed by comparing melt curves against the species-specific qPCR standards, and verified by sequencing as described above. We also tested for inhibition of qPCR by spiking the 10^3^-copy qPCR standard with eDNA samples from aquaria.

### Cost comparison for detection of target species through traditional survey methods

We used two years of mark-recapture data from a long-term turtle conservation and research project in southwestern Ontario to determine the approximate amount of capture effort required to detect the presence of each species at two sites, assuming an extremely low cost of $10 CDN hour of survey effort (the current minimum wage in Ontario is $11.00 CDN). These data spanned two years of surveys at each site, including trapping surveys (hoop traps baited with sardines) and visual surveys (wading in shallow areas and canoeing in deep areas). In practice, the full cost of such surveys includes cost of equipment, travel to survey sites, etc., and will vary among sites, countries and organizations. Our goal was to provide a simplified illustration of variation in detection costs among species, rather than a generalizable quantitative estimate of detection costs. Accordingly, travel time and costs were not included, and we restricted our analysis to a simplified estimate of the cost of surveyor and trapping effort.

We compared the cost of surveyor effort required to detect each species to the cost of eDNA extraction, amplification and sequencing for a single species from a single 1L water sample. Approximate eDNA costs, including labour, were CDN $41.50 using traditional PCR or CDN $43.46 using qPCR. For simplicity we considered the cost of eDNA testing to be approximately CDN $50 per 1L sample. For simplicity, we assumed a sampling effort of 10 water samples for eDNA testing per site, resulting in an estimated cost of $500 CDN to detect a single species at a single site using eDNA. It is expected that costs for actual field studies would vary depending on sample complexity, numbers of target species and PCR replicates required for statistical power, and multiplex assays versus single species detection.

## Results

### Primer specificity

Amplification of *G*. *insculpta* with universal primers was successful, but in general amplification of DNA from turtle blood was inconsistent and problematic with the Universal and Fish COI primers that we tested. Turtle blood samples amplified successfully with AmphF2 and AmphR2, with the exception of *Glyptemys insculpta*. Sequence identity was confirmed in all cases. All nine species-specific primer sets successfully amplified their target species (confirmed by sequencing), and these primers did not cross-amplify any non-target species. COI sequences for most species matched existing conspecific barcode sequences in GenBank (S1 Table). COI sequences for *Chrysemys picta* matched both subspecies found in Canada (*C*. *p*. *marginata* and *C*. *p*. *bellii*). The exception was COI sequences for the *Sternotherus odoratus* (GENBANK accession numbers KM821165 and KM821166). This species was not previously represented in GENBANK or BOLD, and our sequences showed 93 and 94% similarity to *Sternotherus carinatus*.

### eDNA amplification

All nine species were successfully detected from the aquarium water samples through PCR, and negative controls (pre- and post-filtering) showed no evidence of contamination. DNA from *T*. *scripta* was also successfully amplified in all PCR replicates for the 1L sample taken from a small pond. Sequence confirmation of amplified eDNA was successful for all nine species. *Clemmys guttata* and the *Ch*. *serpentina* sample from the high biomass sample were successfully detected using qPCR (Table 2), although the low-biomass *Ch*. *serpentina* sample was not. We did not find evidence of qPCR inhibition in the *C*. *guttata* eDNA sample. Amplification of the quantified control sample (10^3^ target DNA copies/μL) spiked with *Ch*. *serpentina* tank samples showed evidence of inhibition based on reduced product DNA yields at serial dilutions (4.6% amplification of the quantified amount at 1:10 dilution; 8.2% amplification at 1:20 and 83% amplification at 1:30). The dilution replicates also showed some evidence of inhibition: PCR yields for both of the *Ch*. *serpentina* samples which detected the target amplicons plateaued at a dilution of 1:20.One of the *C*. *guttata* sample showed increased yield with each dilution, while the other showed almost equal copy number concentration detected for each dilution.

### Cost comparison of traditional survey methods and eDNA testing

**Fig 1:**
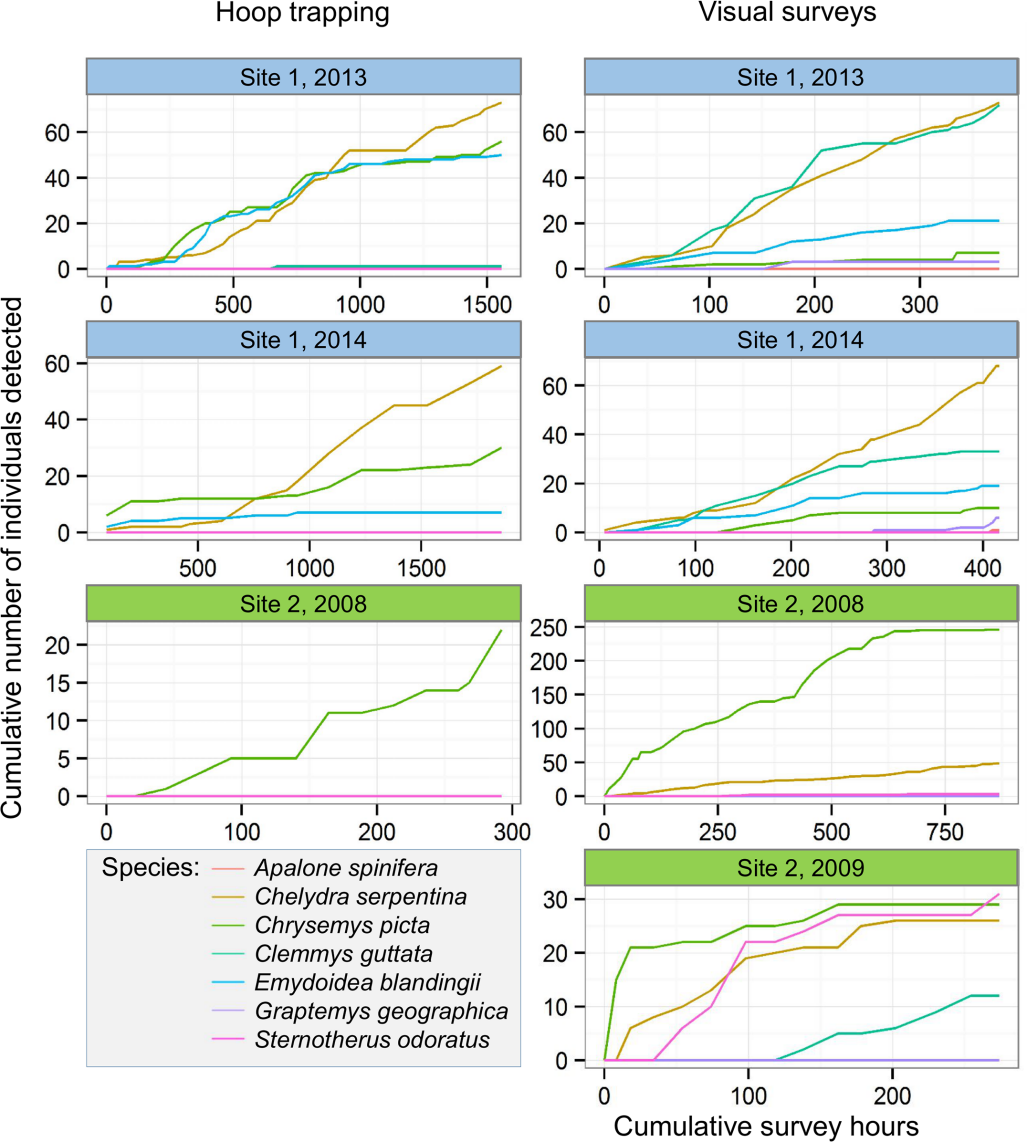
Cumulative detection rates of seven turtle species at two sites where all seven are known to occur, showing substantial spatial and temporal variation. Site 1 (blue) = Lake Erie site, Site 2 (green) = Lake Huron site. Visual surveys include wading and canoe surveys; trapping was done with baited hoop traps. Survey years are indicated in figure captions.

Compared to our simplified cost estimate of $500.00 to detect eDNA from a single turtle species at a site (assuming that detection would be successful), the cost to detect species through traditional field surveys ranged from $100 to $4,100 per detected species (Table 3), and some species were not detected using either trapping or visual surveys (Table 3, Fig 1). Detection rates based on traditional methods varied widely between years and between the two sites. The undetected species were confirmed to be present at these sites in the surveyed years through nesting surveys at known nesting areas ([24]; unpublished data). However, this approach can only be applied once such sites have been identified.

**Table 3:** Approximate hours of survey effort required to detect six of the target species using two traditional survey methods, and estimated labour cost of detection (based on salary of $10 CDN per hour) based on data from two field sites where species presence is known. Cost is based on the number of hours required to detect each species in two consecutive years of intensive survey effort at each site. ND: known to be present at the site, but not detected. NR: never recorded from the site.

		*Hoop trapping*		*Visual surveys*	
Site	Species	Hours until detection (year 1; year 2)	Cost of detection (year 1, year 2)	Hours until detection (year 1, year 2)	Cost of detection (year 1, year 2)
**Lake Erie Site**	*Emydoidea blandingii*	10; 46	$100; $460	18; 38	$180; $380
Trap hours: (1,157; 1,859) Visual survey hours:	*Clemmys guttata*	669; ND	$6,690;—	12; 38	$120; $380
	*Chrysemys picta*	130; 15	$1,300; $150	64; 162.5	$640; $1,625
	*Graptemys geographica*	ND; ND	—	178.3; 286.5	$1,783; $2,865
	*Chelydra serpentina*	37; 92	$370; $920	7; 5	$70; $50
(375; 417)	*Apalone spinifera*	ND; ND	—	ND; 410	—; $4,100
**Lake Huron Site**	*Emydoidea blandingii*	ND	—	ND; ND	—
Trap hours: (292; no year 2) Visual survey hours:	*Clemmys guttata*	ND	—	ND; 138	NR; $1,380
	*Chrysemys picta*	44	$440	8; 10	$80; $100
	*Graptemys geographica*	ND	—	ND; ND	—
	*Chelydra serpentina*	ND	—	22; 18	$220; $180
(869; 274)	*Sternotherus odoratus*	ND	—	274; 54	$2,740; $540
	*Apalone spinifera*	ND	—	ND; 410	—; $4,100

## Discussion

We have demonstrated that turtle DNA can be detected in water samples using appropriate primers, and that eDNA surveys could provide a cost-effective alternative to the variable outcomes of traditional detection methods for freshwater turtles. Field detection rates for our target species vary widely, while species-specific primers successfully identified each target species in small (1L) water samples. The successful detection of *T*. *scripta* in our field sample shows the potential for eDNA sampling in wild turtle habitats. Species that are difficult to detect due to cryptic behaviour (e.g. *A*. *spinifera*) but not due to low population density may be especially good candidates for eDNA surveys. Field tests are now required to assess variation in turtle eDNA detection among sites with different species composition, population densities and hydrological characteristics, and to determine optimal field sampling methods for detection of turtle eDNA.

Although our results demonstrate that eDNA can be applied to detect the occurrence of freshwater turtles under natural conditions, pilot testing to determine how environmental factors may affect eDNA detections is worthwhile and strongly advised. Successful detection of eDNA in aquaria samples does not guarantee success in aquatic habitats, where eDNA concentrations are likely to be much lower. Although we were able to detect *T*. *scripta* in our field sample, eDNA amplification is likely to vary among habitats depending on the abundance of the target species, rates of water flow, and the presence of inhibitors that can prevent the amplification of target DNA [25, 26, 27]. Temporal variation in the rate at which DNA is shed into the environment can also affect the outcome of eDNA surveys in the wild, as can other factors such as UV radiation, temperature, pH and flow rate [1, 25, 28, 29]. Low population densities can limit eDNA detection, and rigorous field trials are required to determine the threshold abundance of target turtle species required for effective eDNA detection at natural sites. The combination of demonstrated PCR inhibition and a lack of detection in the low biomass *Ch*. *serpentina* sample despite known species presence illustrates the need for advance optimization to balance sensitivity and accuracy before initiating field studies. Optimization may include exploring options such as increasing the number of PCR cycles to increase sensitivity, which is common in low yield samples such as eDNA [30]. Dilution of extracted DNA to mitigate inhibition may also require a higher number of PCR cycles to offset low initial target copy number [26]. If increased cycles are necessary, it is advisable to sequence all putative positive results, as increasing cycle numbers has been shown to increase the amount and complexity of nonspecific background products as well as detecting trace contamination, which could both lead to false positives and an inability to confirm results by sequencing [31, 32].

False negatives in presence-absence data can prevent effective habitat protection for threatened species. Failure to detect a species’ presence is more likely where the target species is rare, so smaller populations are less likely to be detected. However, occurrences where the species is rare may be more strongly affected by habitat modifications related to urbanization or natural resources extraction, because increased mortality has a greater effect on small populations [33]. Future work focusing on optimizing field methods for turtle eDNA collection, further testing primer specificity through tests of samples containing multiple species’ DNA, and developing primers targeting other turtle communities could greatly improve detection rates of rare species or focus sampling efforts in areas targeted for development.

Our preliminary comparison of the relative costs of eDNA and traditional turtle survey methods suggests that optimized eDNA surveys may be the most cost-effective approach for documenting species presence, although costs would increase with sample and PCR replicates as well as the number of species being tested for. Costs will also vary among laboratories, methods [34], and sample complexity, as potential difficulties associated with DNA extraction or counteracting PCR inhibitors could require additional steps. Lab costs could also be reduced by using multiplex PCR assays to test for multiple species in a single reaction. Accordingly, rigorous optimization is highly recommended before implementing eDNA detection or monitoring programs. Following optimization, multiple samples would be tested from a single site to assess the likely presence of a species, either by individual species testing or a multiplex assay. As the number of eDNA samples (sample size) and PCR test replicates will vary among studies depending on the research question or goal, desired statistical power, or occupancy modelling, costs for individual studies will vary proportionately. It is strongly recommended that advance planning for sampling should include cost estimates that incorporate travel and labour costs, as well as costs associated with sampling design and detection probabilities (sampling effort, number of replicate samples, sample processing, DNA extraction, and PCR replicates) and desired statistical power. It is also worth noting that eDNA surveys cannot replace some types of information obtained from conventional field methods, such as numbers and size distribution of individuals.

Effective eDNA surveys for turtles could greatly improve the accuracy of population monitoring for threatened turtle species by providing more accurate occupancy data, and identifying areas where target species are present that can then be prioritized for traditional surveys. Species whose conservation could be more effectively achieved with application of eDNA methods include several of the 25 “Most Threatened” [9]. For example, *Rafaetus swinhoei* (Yangtze giant softshell turtle) is reduced to only a few known, captive individuals and may become functionally extinct without successful, rapid intervention through captive breeding [9]. Rapid detection of sites where remaining wild individuals of this species may be present would facilitate targeted capture effort for fertile males, and increase the chance of successful captive breeding of the single known female, or the chance of capturing another female. Similarly, *Dermatemys mawii* (the Central American river turtle) has an extremely restricted range in Belize and south-eastern Mexico, where it is threatened by illegal harvest but is difficult to detect with traditional survey methods [12, 13]. Rapid identification of sites where this species occurs would help prioritize areas for enforcement of existing protective legislation and for education programs targeting consumers of turtle meat.

In Canada, federal and provincial conservation legislation provides protection to the habitat of at-risk species in many areas. However, this legislation does not apply until the species’ presence at a site (habitat) is confirmed. Our field data demonstrate that the amount of survey effort required to detect some species may be prohibitive, and that even with hours of intensive visual and trapping surveys, some species may go undetected (Table 3). Thus, some turtle species such as *C*. *guttata* and *E*. *blandingii* may in fact be more widely distributed than current occurrence records indicate. Recent genetic analyses indicate recent or ongoing genetic connectivity among *C*. *guttata* populations located hundreds of kilometres apart, with no known extant populations or occurrences connecting them [35]. This suggests that undetected occurrences may still exist, and that *C*. *guttata* may be more widespread and therefore less fragmented in their Canadian range than occurrence data indicate. Similar patterns in *E*. *blandingii* [36] also indicate the possible existence of unknown occurrences.

In areas where invasive turtle species are established, eDNA surveys could help track the expansion of these species’ distributions. For example, the red-eared slider (*Trachemys scripta*) is endemic to the eastern and central United States but has been introduced to 18 countries on four continents [37]. In some locations competition with native species constitutes a potential threat to their persistence [38], while in others they have a strong impact as introduced predators of native amphibians [39]. The distribution of this species could be tracked through areas of interest using eDNA, providing a better understanding of its range limitations and habitat requirements. This approach has been used to track a variety of invasive species including Burmese Pythons, Asian carp and American Bullfrog [4, 5, 40].

Looking to the future, turtles’ imperilled status predicts that many species will exhibit significant changes in range and habitat occupancy as landscape modifications and other pressures continue. Replicated eDNA sampling of a number of sites over time can be used to track these shifts, and to test specific hypotheses about the factors that influence persistence or extinction in populations of different species. These questions are typically not well suited to long-lived organisms such as turtles, especially given the challenges around their detection in the field and acknowledged concerns of detection failure. Using eDNA surveillance to augment traditional survey methods will not eliminate risk associated with detection failure, but can reduce the incidence of false negative results and help identify areas that may merit greater field efforts. As such, targeted eDNA surveys present an opportunity to cost-effectively document changes in community structure over time in suites of long-lived, iteroparous organisms, and contribute to their effective conservation.

## Supporting Information

S1 Table Accession numbers for cytochrome oxidase 1 (CO1) sequences used to design environmental DNA (eDNA) primers for the nine target species. The matrix shows the number of mismatches (nucleotide substitutions) between intraspecific consensus sequences for each pair of target and non-target species within a trimmed 675bp alignment.
(DOCX)
