# Supplementary material for: Development and Validation of Environmental DNA (eDNA) Markers for Detection of Freshwater Turtles
Source: PLoS One. 2015 Jul 22;10(7):e0130965. doi: 10.1371/journal.pone.0130965 (PMC4511736; doi:10.1371/journal.pone.0130965)
Supplement: S1 Table — The matrix shows the number of mismatches (nucleotide substitutions) between intraspecific consensus sequences for each pair of target and non-target species within a trimmed 675bp alignment. (DOCX) [file pone.0130965.s001.docx]

**Supplemental Table 1.** Accession numbers for cytochrome oxidase 1 (CO1) sequences used to design environmental DNA (eDNA) primers for the nine target species. The matrix shows the number of mismatches (nucleotide substitutions) between intraspecific consensus sequences for each pair of target and non-target species within a trimmed 675bp alignment.

|  | BOLD | Genbank | Non-target species | | | | | | | | |
| --- | --- | --- | --- | --- | --- | --- | --- | --- | --- | --- | --- |
| Target species  and accession numbers |  |  | *Emydoidea blandingii* | *Clemmys guttata* | *Glyptemys insculpta* | *Chrysemys picta* | *Graptemys geographica* | *Sternotherus odoratus* | *Chelydra serpentina* | *Apalone spinifera* | *Trachemys scripta* |
| *Emydoidea blandingii* | BENT126-08  GBGC11274-13  GBGC11445-13 | N/A  HQ329642  KC181200 | -- | 52 | 48 | 65 | 75 | 76 | 99 | 128 | 80 |
| *Clemmys guttata* | BENT113-08  BGC11275-13  GBGC11396-13  GBGC11444-13 | N/A  HQ329641  KC750819  KC181201 |  | -- | 54 | 73 | 79 | 77 | 100 | 121 | 80 |
| *Glyptemys insculpta* | BENT110-08  GBGC11272-13  GBGC11392-13  GBGC11438-13 | N/A  HQ329644  KC750823  KC181207 |  |  | -- | 71 | 73 | 85 | 101 | 120 | 79 |
| *Chrysemys picta* | CYTC4100-12  GBGC1504-06  GBGC11164-13  GBGC11436-13 | AF069423  NC_002073  JN993981  KC181209 |  |  |  | -- | 49 | 89 | 91 | 132 | 51 |
| *Graptemys geographica* | GBGC11162-13  GBGC11163-13 | JN993983  JN993982 |  |  |  |  | -- | 90 | 101 | 129 | 28 |
| *Sternotherus spp*  ***  **** | BENT287-09  CYTC3823-12  CYTC4973-12  GBGC11232-13 | N/A  NC_017607  HQ114563  HQ329724 |  |  |  |  |  | -- | 91 | 114 | 93 |
| *Chelydra serpentina* | CYTC4506-12  GBGC1313-06  GBGC7693-09  GBGC11393-13 | EF122793  DQ256378  NC_011198  KC750822 |  |  |  |  |  |  | -- | 126 | 104 |
| *Apalone spinifera* | GBGC12687-13  GBGC12953-13 | NC_021371  JF966197 |  |  |  |  |  |  |  | -- | 130 |
| *Trachemys scripta* | GBGC1819-06  GBGC6907-09  GBGC6939-09  GBGC10659-13 | U49047  NC_011573  FJ392294  JF700194 |  |  |  |  |  |  |  |  | -- |

* Note 1: As no CO1 sequence for *Sternotherus odoratus* were available in BOLD or GenBank, CO1 sequences for congeneric species were used for primer design, including *Sternotherus depressus* and *Sternotherus carinatus* sequences.

* * Note 2: Inter specific differences are estimates based on consensus *Sternotherus species* and should be considered as approximate.
